# The aryl sulfonamide indisulam inhibits gastric cancer cell migration by promoting the ubiquitination and degradation of the transcription factor ZEB1

**DOI:** 10.1016/j.jbc.2023.103025

**Published:** 2023-02-15

**Authors:** Jiaqi Lu, Dan Li, Honglv Jiang, Yue Li, Chengpiao Lu, Tao Chen, Yuhong Wang, Xiaohui Wang, Wenzhao Sun, Zhongjian Pu, Chunhua Qiao, Jingjing Ma, Guoqiang Xu

**Affiliations:** 1 Jiangsu Key Laboratory of Neuropsychiatric Diseases and College of Pharmaceutical Sciences, Jiangsu Province Engineering Research Center of Precision Diagnostics and Therapeutics Development, Jiangsu Key Laboratory of Preventive and Translational Medicine for Geriatric Diseases, Suzhou Key Laboratory of Drug Research for Prevention and Treatment of Hyperlipidemic Diseases, Soochow University, Suzhou, Jiangsu, China; 2 Department of General Surgery, The First Affiliated Hospital of Soochow University, Suzhou, Jiangsu, China; 3 Department of Pathology, The First Affiliated Hospital of Soochow University, Suzhou, Jiangsu, China; 4 Department of Oncology, Haian Hospital of Traditional Chinese Medicine, Haian, Jiangsu, China; 5 College of Pharmaceutical Sciences, Soochow University, Suzhou, Jiangsu, China; 6 Department of Pharmacy, Medical Center of Soochow University, Dushu Lake Hospital Affiliated to Soochow University, Suzhou, Jiangsu, China

**Keywords:** indisulam, label-free quantification, quantitative proteomics, N-cadherin, DCAF15, ZEB1, ubiquitination, degradation, migration

## Abstract

Gastric cancer is one of the cancers with high morbidity and mortality worldwide. The aryl sulfonamide indisulam inhibits the proliferation of several types of cancer cells through its function as a molecular glue to promote the ubiquitination and degradation of RNA-binding motif protein 39 (RBM39). However, it is unknown whether and how indisulam regulates the migration of cancer cells. In this work, using label-free quantitative proteomics, we discover that indisulam significantly attenuates N-cadherin, a marker for epithelial to mesenchymal transition and migration of cancer cells. Our bioinformatics analysis and biochemical experiments reveal that indisulam promotes the interaction between the zinc finger E-box-binding homeobox 1 (ZEB1), a transcription factor of N-cadherin, and DCAF15, a substrate receptor of CRL4 E3 ubiquitin ligase, and enhances ZEB1 ubiquitination and proteasomal degradation. In addition, our cell line–based experiments demonstrate that indisulam inhibits the migration of gastric cancer cells in a ZEB1-dependent manner. Analyses of patient samples and datasets in public databases reveal that tumor tissues from patients with gastric cancer express high *ZEB1* mRNA and this high expression reduces patient survival rate. Finally, we show that treatment of gastric tumor samples with indisulam significantly reduces ZEB1 protein levels. Therefore, this work discloses a new mechanism by which indisulam inhibits the migration of gastric cancer cells, indicating that indisulam exhibits different biological functions through distinct signaling molecules.

Gastric cancer is among the five types of cancers that have the highest morbidity and mortality (1). Although the treatment for gastric cancer has been improved significantly in the past decades (2), the overall patient survival rate is still not satisfactory due to metastasis, drug resistance, side reaction, etc. (3). Inhibition of proliferation, migration, and invasion of cancer cells is an important strategy for cancer treatment. Therefore, identifying molecules that regulate these processes or elucidating the mechanism of action of anticancer drugs could help us to develop new therapeutic strategies for targeted cancer treatment.

Epithelial to mesenchymal transition (EMT) is associated with the metastasis of many cancers, and its activation promotes the migration, invasion, drug resistance, and recurrence of cancer cells (4, 5). The main characteristics for EMT are the reduction of epithelial markers such as cell adhesive molecule E-cadherin and the upregulation of mesenchymal markers including N-cadherin, vimentin, and β-catenin. EMT is regulated by many transcription factors such as zinc finger E-box-binding homeobox 1/2 (ZEB1/2), zinc finger protein SNAI1 (Snail) and SNAI2 (Slug), and twist-related protein 1 (Twist) (6, 7, 8). ZEB1 is highly expressed in tumor tissues of multiple cancers including neuroblastoma, lung cancer, breast cancer, and pancreatic cancer, and its expression is positively correlated with the invasiveness of cancer cells (9, 10, 11, 12, 13). High expression of ZEB1 is accompanied with the reduced expression of E-cadherin and enhanced expression of N-cadherin (14), thus reducing the adhesion of epithelial cells and promoting the migration, metastasis, and invasion, leading to the poor prognosis (15, 16). It was reported that ZEB1 itself was regulated by the ubiquitin-proteasome system (UPS). Recently, high-throughput proteomic data discovered that ZEB1 interacted with DDB1- and Cul4-associated factor 15 (DCAF15) (17, 18, 19), a substrate receptor of a cullin 4-RING E3 ubiquitin ligase (CRL4), which also consists of DNA damage-binding protein 1 (DDB1), cullin 4A/B, and RING box protein (Rbx1 or Roc1) (20, 21). Thus, DCAF15 promotes the ubiquitination and degradation of ZEB1 and negatively regulates its protein level, thereby suppressing malignancy of hepatocellular carcinoma (HCC) (22). However, it is not clear whether this regulation occurs in other cancer cells and is responsible for the tumor cell proliferation and metastasis.

It has been reported that aryl sulfonamides such as indisulam (E7070), E7820, and tasisulam (LY573636) could inhibit the proliferation of several types of cancer cells through different signaling pathways (23, 24, 25, 26), although the direct molecular target was unknown until 5 years ago (27). Detailed mechanistic studies revealed that these compounds act as molecular glues to initiate the interaction between DCAF15 and the neosubstrate RNA-binding protein RBM39, which was subsequently ubiquitinated and degraded by the 26S proteasome. Thereby, these compounds exhibit their anticancer activity by inhibiting the proliferation of cancer cells (27, 28, 29, 30, 31). However, it is unknown whether and how these compounds affect the migration of gastric cancer cells.

In this work, we use label-free quantitative proteomics to screen proteins that are differentially regulated by indisulam in a gastric cancer cell line and discover that the marker protein in the EMT, N-cadherin, is significantly attenuated. Scratch assay further reveals the effect of indisulam on the migration of gastric cancer cells. Analysis of Biological General Repository for Interaction Datasets (BioGRID) uncovers that DCAF15 interacts with ZEB1, a key transcription factor regulating EMT and migration of cancer cells. Biochemical experiments disclose that indisulam downregulates ZEB1 through the UPS in a DCAF15-dependent manner. Scratch assay and small RNA interference reveal that ZEB1 is critical for the inhibitory effect of indisulam on the migration of gastric cancer cells. Finally, clinical samples are utilized to validate the mechanism discovered in the cell line–based experiments.

## Results

### Label-free quantitative proteomics identified indisulam-downregulated protein that regulates cell migration

**Figure 1:**
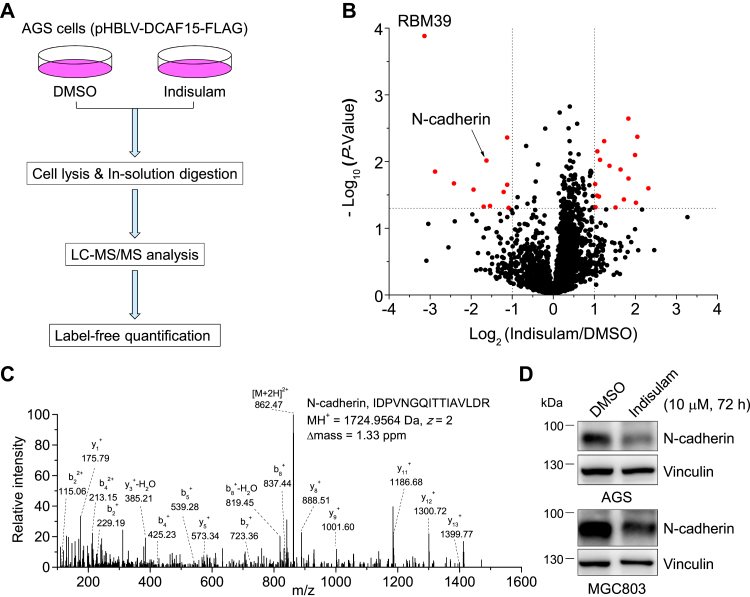
**Quantitative proteomics and biochemical approaches discover and verify that indisulam downregulates N-cadherin in gastric cancer cells.***A*, flowchart for the label-free quantitative proteomics used in this work. *B*, volcano plot for proteins identified by proteomics analysis of cell lysates obtained from the DMSO- or indisulam-treated (10 μM, 6 h) AGS cells expressing DCAF15. −Log_10_ (*p*-value) and Log_2_ (Indisulam/DMSO) were obtained from Proteome Discoverer database search and Perseus analysis. Proteins (*red circles*) with −Log_10_ (*p*-value) >1.30 (*i.e.*, *p*-value < 0.05, *horizontal dotted line*) and Log_2_ (Indisulam/DMSO) >1.0 or <−1.0 (*vertical dotted lines*) were considered as differentially regulated by indisulam. *C*, tandem mass spectrometry spectrum of a representative tryptic peptide derived from N-cadherin. The amino acid sequence, charge state (*z*), MH^+^, and Δmass were provided. *D*, Western blotting analysis of cell lysates obtained from AGS and MGC803 cells treated with DMSO or indisulam (10 μM) for 72 h. DMSO, dimethyl sulfoxide.

To explore the potential biological processes that indisulam might regulate in gastric cancer cells, we used label-free quantitative (LFQ) proteomics to identify proteins that are differentially regulated by indisulam following the procedure described in Figure 1*A*. Briefly, AGS cells expressing DCAF15 from a previous work (32) were treated with dimethyl sulfoxide (DMSO) (control) or indisulam and lysed. Proteins in cell lysates were reduced, blocked, digested, desalted, and analyzed by Orbitrap Fusion Lumos Tribrid mass spectrometer. Database search with Proteome Discoverer and quantification with LFQ and Perseus for data from three biological replicates obtained 3340 quantified proteins (Table S1). Among them, 28 proteins were considered as differentially regulated by indisulam, all of them with −Log_10_ (*p*-value) >1.30 (*i.e.*, *p*-value < 0.05) and Log_2_ (Indisulam/DMSO) >1.0 or <−1.0 (Fig. 1*B* and Table S2). Consistent with previous discoveries (32), RNA-binding motif protein 39 (RBM39) is the most significantly downregulated protein. It has been reported previously that RBM39 mediates the indisulam-induced cytotoxicity in several types of cancer cells (27) and the indisulam-inhibited proliferation of gastric cancer cells (32). Therefore, we focused on other potential targets.

Interestingly, cadherin-2 (encoded by *CDH12*), also called as N-cadherin, was among the most significantly downregulated proteins, with a −Log_10_ (*p*-value) of 2.01 and Log_2_ (Indisulam/DMSO) of −1.63 (Fig. 1*B*, indicated by the black arrow). Mass spectrometry (MS) discovered two tryptic peptides derived from N-cadherin, with a sequence coverage of 4.6%. The tandem mass spectrometry spectrum of a representative peptide verified the confident identification of this protein, indicated by the high-quality matches between the experimental and theoretical *b*- and *y*-ions (Fig. 1*C*). Western blotting analysis further confirmed that N-cadherin was indeed attenuated by indisulam (Fig. 1*D*). To explore how indisulam regulates N-cadherin, we treated AGS cells with protein synthesis inhibitor cycloheximide (CHX) and discovered that indisulam did not significantly alter the degradation of N-cadherin (Fig. S1*A*). However, quantitative PCR revealed that indisulam downregulated the *N-cadherin* mRNA level (Fig. S1*B*). These data clearly demonstrated that indisulam downregulated N-cadherin through reducing its gene expression.

### Indisulam inhibits the migration of gastric cancer cells

**Figure 2:**
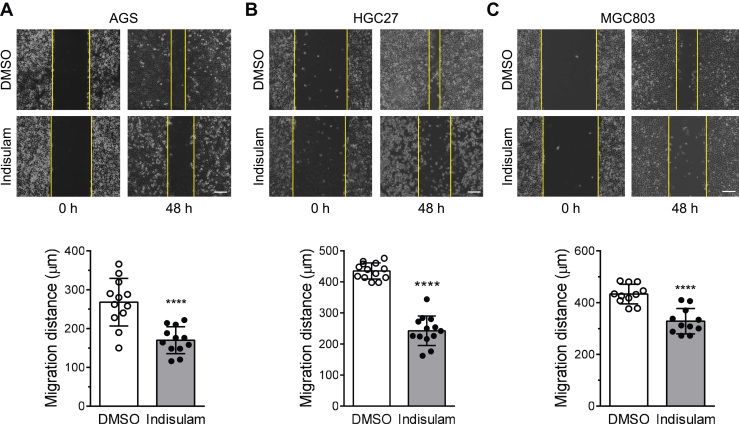
**Indisulam inhibits the migration of gastric cancer cells.** Scratch assay was utilized to evaluate the relative migration of gastric cancer cells AGS (*A*), HGC27 (*B*), and MGC803 (*C*). Representative images were obtained from cells treated with DMSO or indisulam (10 μM) for 0 and 48 h. The *vertical yellow lines* indicated the edges of the scratch. Bar graphs showed the mean and standard deviations (mean ± SD, n = 13, 12, and 11, respectively, from three biological replicates). Student’s *t* test, ∗∗∗∗*p* < 0.0001. The scale bar represents 200 μm. DMSO, dimethyl sulfoxide.

Abnormal proliferation, metastasis, and invasion are major features of the progression of gastric cancer. Therefore, drugs that suppress these processes may have potentials for the therapeutic treatment of gastric cancer. Our previous studies have discovered that indisulam can significantly inhibit the proliferation of gastric cancer cells by promoting the ubiquitination and degradation of RBM39 (32). Among the identified proteins, N-cadherin is a marker for EMT and migration of cancer cells (33). Therefore, we sought to test whether indisulam affects the migration of gastric cancer cells using a scratch assay. Measurement of migration distance for three gastric cancer cell lines (HGC27, AGS, and MGC803) disclosed that indisulam could significantly reduce the migration of gastric cancer cells (Fig. 2, *A*–*C*).

### *DCAF15* knockdown eliminates the inhibitory effect of indisulam on the migration of gastric cancer cells

**Figure 3:**
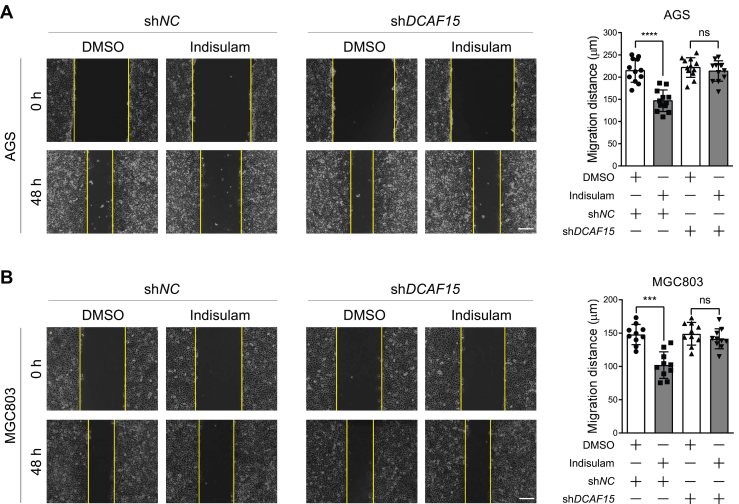
***DCAF15* knockdown eliminates the inhibitory effect of indisulam on the migration of gastric cancer cells.***A* and *B*, scratch assay was performed for sh*NC* and sh*DCAF15*-expressing AGS and MGC803 cells in the presence of dimethyl sulfoxide (DMSO) or indisulam (10 μM). Representative images were taken at 0 h and 48 h after treatment. *Vertical yellow lines* indicated the edge of the scratch. Mean ± SD (n = 12 or 10 from three biological replicates), Student’s *t* test, ∗∗∗*p* < 0.001, ∗∗∗∗*p* < 0.0001, ns: not significant. The scale bar represents 200 μm.

Previous studies have demonstrated that indisulam could promote RBM39 degradation through the UPS and thus mediate the cytotoxicity to cancer cells (27, 28, 29). It was also manifested that indisulam induced the interaction between RBM39 and DCAF15, the substrate receptor of Cullin 4-RING E3 Ligase (CRL4), which was required for the indisulam-induced RBM39 degradation. Therefore, we thought to test whether DCAF15 is also required for the inhibitory effect of indisulam on the migration of gastric cancer cells. For this, we knocked down *DCAF15* in AGS and MGC803 cells (Fig. S2, *A* and *B*) and treated them with DMSO and indisulam for the scratch assay. The results showed that *DCAF15* knockdown abolished the inhibitory effect of indisulam on the migration of AGS and MGC803 cells (Fig. 3, *A* and *B*), indicating that DCAF15 played a critical role in this regulation.

### Indisulam enhances the interaction between ZEB1 and DCAF15

**Figure 4:**
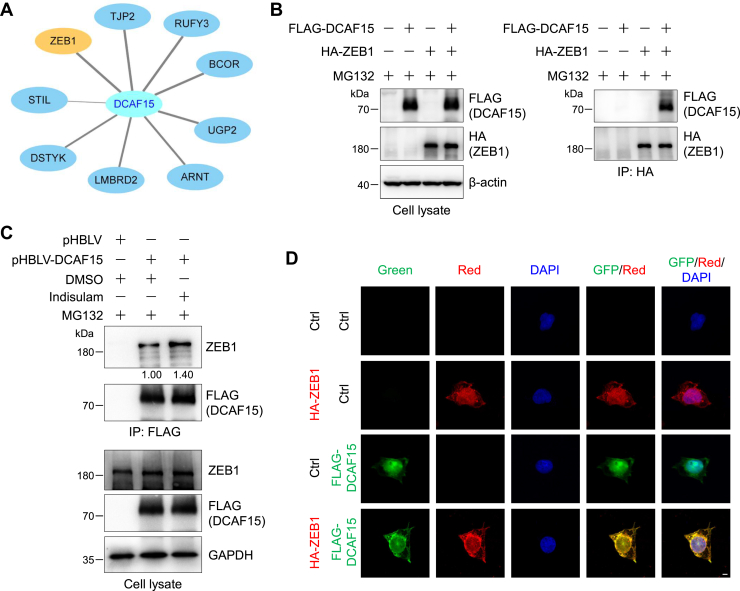
**Indisulam enhances the interaction between ZEB1 and DCAF15.***A*, DCAF15-interacting proteins were obtained from BioGRID (https://thebiogrid.org/). The cutoff threshold for the modified CompPASS score (https://bioplex.hms.harvard.edu/comppass/) is >0.75. The score for ZEB1 is 1.00. *B*, ZEB1 interacts with DCAF15. HEK293T cells were transfected with FLAG-DCAF15 and/or hemagglutinin (HA)-ZEB1 plasmids and then split into two plates for 48 h. Cells were pretreated with MG132 (10 μM) for 2 h and lysed. ZEB1 was immunoprecipitated with anti-HA magnetic beads. Cell lysates and immunoprecipitates were subjected to immunoblotting analysis. *C*, indisulam enhances the DCAF15-ZEB1 interaction. FLAG-DCAF15 plasmid or control vector was transfected into HEK293T cells for 48 h. Cells were pretreated with MG132 (10 μM) for 2 h and then treated with dimethyl sulfoxide (DMSO) or indisulam (10 μM) for 14 h. Cells were lysed, and DCAF15 and its interacting proteins were immunoprecipitated with anti-FLAG affinity gel. Cell lysates and immunoprecipitates were subjected to immunoblotting analysis. The relative intensity of the immunoprecipitated ZEB1 was shown below the image. *D*, ZEB1 colocalizes with DCAF15 in the nucleus. HEK293 cells were transiently transfected with the control or indicated plasmids for 24 h, fixed, and incubated with the primary and fluorescent secondary antibodies. Immunofluorescence was detected under a confocal microscope. The scale bar represents 5 μm.

Since DCAF15 is required for the inhibitory effect of indisulam on the migration of gastric cancer cells, we thought that DCAF15-interacting proteins may participate in this regulation. To explore this possibility, we searched the BioGRID database and discovered several proteins that interact with DCAF15 with a high level of confidence (Fig. 4*A*) (17, 18, 19). Among them, ZEB1 has been reported to be closely associated with tumor metastasis (34, 35, 36, 37, 38, 39). To verify the interaction between DCAF15 and ZEB1 biochemically, we performed coimmunoprecipitation experiments. Western blotting images disclosed that exogenous ZEB1 interacted with DCAF15 (Fig. 4*B*). Similarly, exogenous DCAF15 interacted with endogenous ZEB1 and indisulam enhanced this interaction by about 40% (Fig. 4*C*). To further validate their interaction, we performed immunofluorescence experiments, which uncovered that DCAF15 and ZEB1 mostly colocalized in the nucleus with the Pearson correlation coefficient of 0.905 ± 0.045 (Figs. 4*D* and S3). Taken together, these results indicated that DCAF15 interacted with ZEB1 and this interaction was enhanced by indisulam.

### Indisulam downregulates ZEB1 in a time- and dose-dependent manner

**Figure 5:**
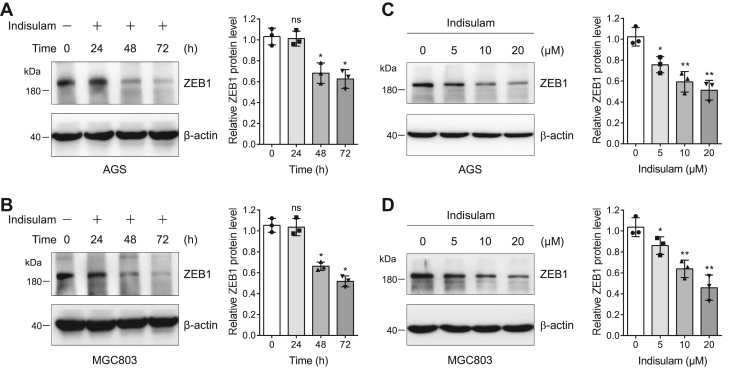
**Indisulam downregulates ZEB1 in a time- and dose-dependent manner.***A* and *B*, AGS and MGC803 cells were treated with indisulam (10 μM) for different times and the resulting cell lysates were subjected to immunoblotting analysis. Mean ± SD (n = 3), Student’s *t* test, ∗*p* < 0.05, ns: not significant. *C* and *D*, AGS and MGC803 cells were treated with dimethyl sulfoxide or different concentrations of indisulam for 72 h. The cell lysates were subjected to immunoblotting analysis. Mean ± SD (n = 3), Student’s *t* test, ∗*p* < 0.05, ∗∗*p* < 0.01.

Since DCAF15 is a substrate receptor that recognizes specific proteins for their ubiquitination and degradation, we thought to examine whether indisulam regulates ZEB1 protein level. We treated AGS and MGC803 cells with DMSO or indisulam for 0, 24, 48, and 72 h and immunoblotted the cell lysates for ZEB1. The results unveiled that indisulam decreased ZEB1 protein gradually in AGS and MGC803 cells with the increase of treatment time (Fig. 5, *A* and *B*). Furthermore, we found that ZEB1 protein level was downregulated progressively with the increase of the indisulam concentration in these two cell lines (Fig. 5, *C* and *D*). These results indicated that indisulam downregulated ZEB1 protein levels in a time- and dose-dependent manner in gastric cancer cells.

### DCAF15 mediates the indisulam-induced downregulation of ZEB1

**Figure 6:**
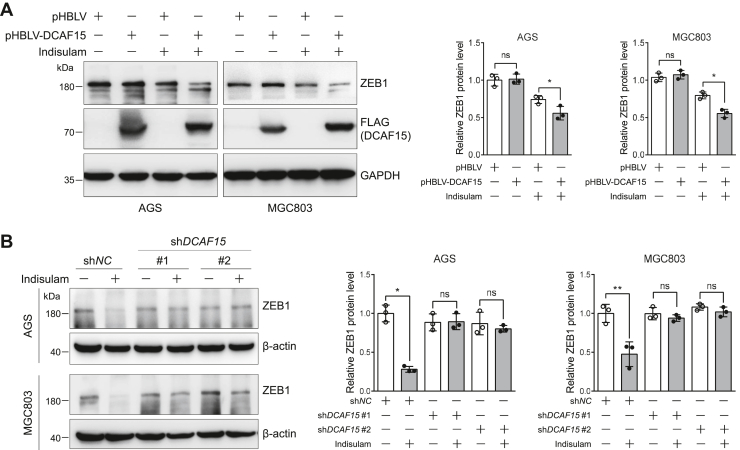
**DCAF15 enhances the indisulam-induced degradation of ZEB1 in gastric cancer cells.***A*, overexpression of DCAF15 accelerates the downregulation of ZEB1 induced by indisulam. The stable pHBLV (control) and pHBLV-DCAF15-expressing AGS and MGC803 cells were treated with dimethyl sulfoxide or indisulam (10 μM) for 48 h. The resulting cell lysates were subjected to immunoblotting analysis. Mean ± SD (n = 3), Student’s *t* test, ∗*p* < 0.05, ns: not significant. *B*, *DCAF15* knockdown abolishes the indisulam-induced downregulation of ZEB1. The stable sh*NC* and sh*DCAF15*-expressing AGS and MGC803 cells were treated with dimethyl sulfoxide or indisulam (10 μM) for 48 h. The resulting cell lysates were subjected to immunoblotting analysis. Mean ± SD (n = 3), Student’s *t* test, ∗*p* < 0.05, ∗∗*p* < 0.01, ns: not significant.

The above results demonstrated that DCAF15 interacted with ZEB1 and indisulam downregulated ZEB1 in gastric cancer cells. The next question we would like to ask is whether the downregulation of ZEB1 by indisulam requires DCAF15. To answer this question, we constructed AGS and MGC803 cells stably expressing control and DCAF15 and verified its expression by immunoblotting (Fig. S4, *A* and *B*). The scratch assay indicated that DCAF15 expression alone did not alter the migration of AGS and MGC803 cells (Fig. S4, *C* and *D*). In addition, gradient transfection of DCAF15 did not affect ZEB1 protein level (Fig. S5). Immunoblotting of cell lysates obtained from cells treated with DMSO or indisulam revealed that DCAF15 expression accelerated the indisulam-induced downregulation of ZEB1 protein (Fig. 6*A*). Consistent with this, *DCAF15* knockdown almost completely abolished the indisulam-induced downregulation of ZEB1 in AGS and MGC803 cells (Fig. 6*B*).

### Indisulam downregulates ZEB1 through the UPS

**Figure 7:**
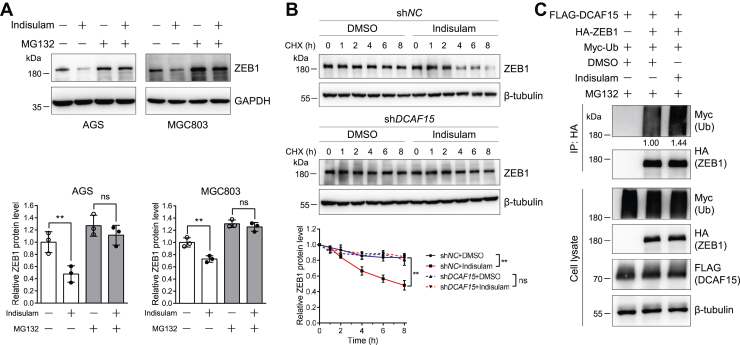
**Indisulam induces ZEB1 degradation through the ubiquitin proteasome system.***A*, proteasome inhibitor MG132 abolishes the indisulam-induced ZEB1 degradation. AGS and MGC803 cells were pretreated with DMSO or indisulam (10 μM) for 48 h and then treated with DMSO or MG132 (10 μM) for 16 h in the presence of DMSO or indisulam. The resulting cell lysates were subjected to immunoblotting analysis. Mean ± SD (n = 3), Student’s *t* test, ∗∗*p* < 0.01, ns: not significant. *B*, *DCAF15* knockdown eliminates the indisulam-induced degradation of ZEB1. AGS cells stably expressing sh*NC* and sh*DCAF15* were treated with DMSO or indisulam (10 μM) and cycloheximide (200 μg/ml) for the indicated time. Mean ± SD (n = 3), two-way ANOVA, ∗∗*p* < 0.01, ns: not significant. *C*, indisulam promotes ZEB1 ubiquitination. HEK293T cells were transfected with the indicated plasmids for 48 h, pretreated with MG132 (10 μM) for 2 h, and treated again with DMSO or indisulam (10 μM) for 12 h in the presence of MG132. ZEB1 was immunoprecipitated with anti-HA magnetic beads. The cell lysates and immunoprecipitates were subjected to immunoblotting analysis. The relative intensity of ubiquitinated ZEB1 was shown below the image. The experiments were performed twice and similar results were obtained. DMSO, dimethyl sulfoxide; HA, hemagglutinin.

Since DCAF15 is a substrate receptor of CRL4 E3 ubiquitin ligase and the reduction of ZEB1 by indisulam requires DCAF15, we analyzed the role of UPS in the indisulam-induced ZEB1 reduction. First, we evaluated the effect of a proteasome inhibitor MG132 on this regulation. Similar to the above discovery, indisulam significantly reduced ZEB1 protein levels in the absence of MG132. However, in the presence of MG132, indisulam no longer reduced ZEB1 in AGS and MGC803 cells (Fig. 7*A*). Second, we investigated the effect of DCAF15 on the degradation rate of ZEB1. The CHX chase experiments uncovered that indisulam accelerated the degradation of ZEB1, which was almost completely abolished by knocking down *DCAF15* (Fig. 7*B*). These results suggest that indisulam-induced degradation of ZEB1 is most probably regulated by the UPS. Third, we explored the effect of indisulam on the ubiquitination of ZEB1. Immunoblotting of whole cell lysates and anti-hemagglutinin (HA) immunoprecipitates unveiled that indisulam could increase ZEB1 ubiquitination by 44% (Fig. 7*C*). In addition, surprisingly, the quantitative PCR (qPCR) experiments showed that indisulam increased the *ZEB1* mRNA level (Fig. S6), further supporting the idea that the degradation of ZEB1 is responsible for the reduction of its protein level. Taken together, these results demonstrated that indisulam degraded ZEB1 through the UPS.

### Indisulam inhibits the migration of gastric cancer cells by degrading ZEB1

**Figure 8:**
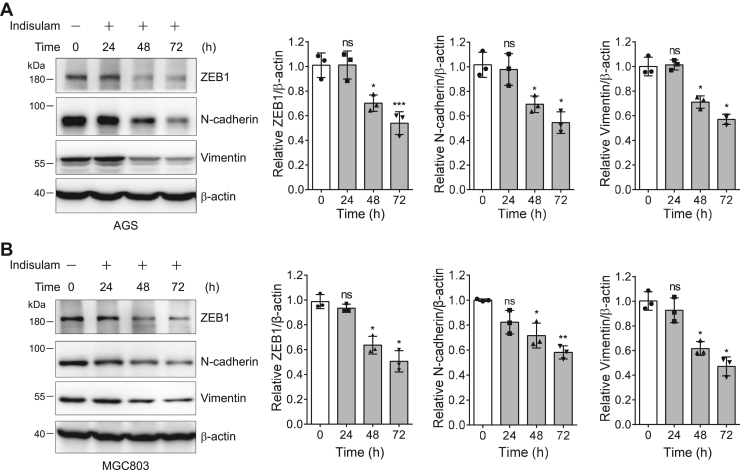
**Indisulam attenuates the epithelial to mesenchymal transition markers in gastric cancer cells.***A* and *B*, the protein level of EMT markers was immunoblotted for cell lysates obtained from gastric cancer cells AGS (*A*) and MGC803 (*B*) treated with indisulam (10 μM) for different time. Mean ± SD (n = 3), Student’s *t* test, ∗*p* < 0.05, ∗∗*p* < 0.01, ∗∗∗*p* < 0.001, ns: not significant.

Many studies have discovered that ZEB1 is involved in the regulation of EMT, which is closely associated with metastasis of tumor cells (40). Therefore, we next asked whether indisulam inhibits the migration of gastric cancer cells by downregulating ZEB1. Western blotting of cell lysates obtained from cells treated with DMSO or indisulam clearly indicated that ZEB1 and two EMT markers (N-cadherin and vimentin) decreased gradually with the increase of indisulam treatment time in AGS and MGC803 cells (Fig. 8, *A* and *B*).

**Figure 9:**
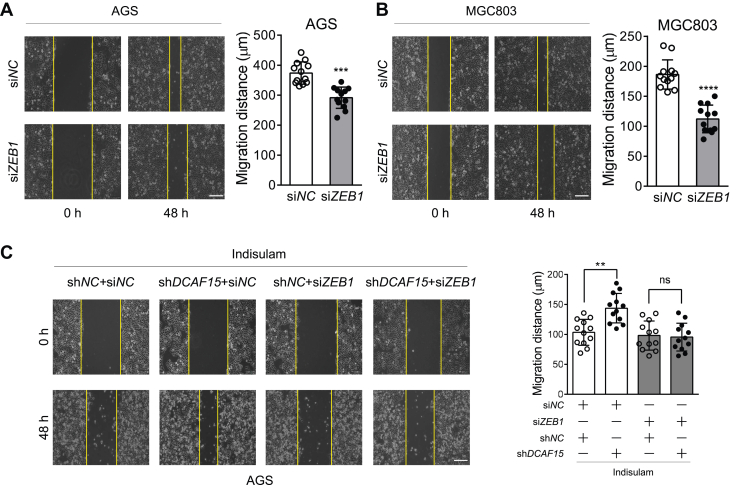
**ZEB1 mediates the indisulam-inhibited migration of gastric cancer cells.***A* and *B*, *ZEB1* knockdown reduces the migration of AGS and MGC803 cells. Cells were transfected with the control and *ZEB1*-specific siRNA with lipofectamine RNAiMAX. At 24 h after transfection, the scratch assay was performed. Mean ± SD (n = 12 from three biological replicates), Student’s *t* test, ∗∗∗*p* < 0.001, ∗∗∗∗*p* < 0.0001. The scale bar represents 200 μm. *C*, *ZEB1* knockdown attenuates the DCAF15-mediated inhibitory effect of indisulam on the migration of AGS cells. The sh*NC* and sh*DCAF15*-expressing stable AGS cells were transfected with the control and *ZEB1*-specific siRNA with lipofectamine RNAiMAX. At 24 h after transfection, the scratch assay was performed in the presence of indisulam (10 μM). Mean ± SD (n = 12 from three biological replicates), Student’s *t* test, ∗∗*p* < 0.01, ns: not significant. The scale bar represents 200 μm.

To further explore the role of ZEB1 on the inhibitory effect of indisulam on the migration of gastric cancer cells, we first carried out the scratch assay with AGS and MGC803 cells in which *ZEB1* was knocked down by siRNA (Fig. S7). The scratch assay revealed that *ZEB1* knockdown indeed inhibited the migration of AGS and MGC803 cells (Fig. 9, *A* and *B*). Then, we transfected si*NC* and si*ZEB1* into AGS cells stably expressing sh*NC* and sh*DCAF15*, respectively. Consistent with the above results, *DCAF15* knockdown eliminated the inhibitory effect of indisulam on the migration of gastric cancer cells. However, after *ZEB1* knockdown, further knockdown of *DCAF15* did not alter the effect of indisulam on the migration of gastric cancer cells (Fig. 9*C*). Moreover, Western blotting analysis confirmed that *ZEB1* knockdown completely blocked the indisulam-induced reduction of EMT markers N-cadherin and vimentin (Fig. S8). Collectively, these data demonstrated that indisulam inhibited the migration of gastric cancer cells by degrading ZEB1 through DCAF15.

### High expression of *ZEB1* reduces the survival time of patients with gastric cancer

**Figure 10:**
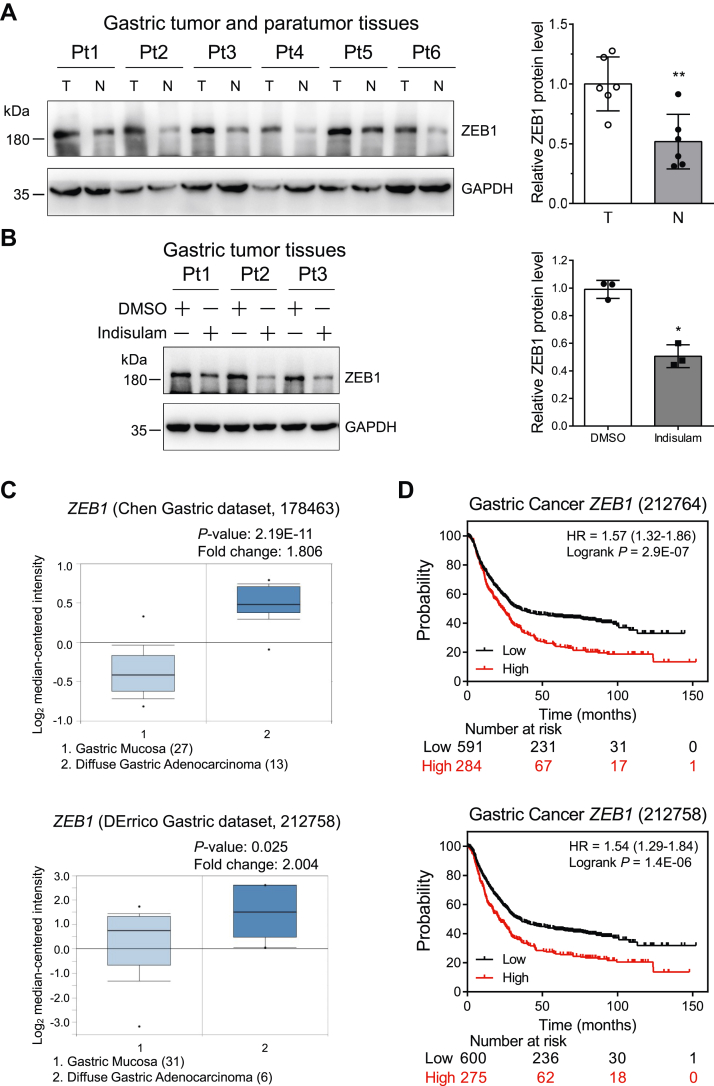
**Indisulam reduces ZEB1 in gastric cancer tissues, and high expression of *ZEB1* decreases the overall survival of patients with gastric cancer.***A*, immunoblotting of ZEB1 in normal (paratumor) and tumor tissues from six patients with gastric cancer. Pt: patient, T: Tumor, N: Normal, Mean ± SD (n = 6), Student’s *t* test, ∗∗*p* < 0.01. *B*, gastric cancer tissues were treated with dimethyl sulfoxide (DMSO) and indisulam, and ZEB1 in tissue lysates was immunoblotted. Mean ± SD (n = 3), Student’s *t* test, ∗*p* < 0.05. *C*, the relative mRNA level of *ZEB1* in gastric mucosa and diffuse gastric adenocarcinoma was obtained from Oncomine (https://www.oncomine.org). *D*, the correlation between the *ZEB1* mRNA and the overall survival of patients with gastric cancer was obtained from Kaplan–Meier plotter (https://kmplot.com/analysis/).

Since our cell line–based experiments discovered that indisulam directly modulated ZEB1, we analyzed the expression level of ZEB1 instead of N-cadherin and DCAF15 in gastric cancer tissues to further explore the role of ZEB1 in the progression of gastric cancer. Immunoblotting analysis uncovered that ZEB1 was expressed higher in gastric cancer tissues than in the paratumor tissues (Fig. 10*A*). To further confirm the regulation of indisulam on ZEB1 in gastric cancer tissues, we used different concentrations of indisulam to treat gastric cancer tissues in culture plates. Immunoblotting of total cell lysates indicated that indisulam downregulated ZEB1 in a dose-dependent manner (Fig. S9). Concordantly, indisulam attenuated ZEB1 protein level in all three tested gastric tumor tissues (Fig. 10*B*). Consistent with the experimental results in cell lines, indisulam also increased the *ZEB1* mRNA level in gastric tumor tissues (Fig. S10). Moreover, analysis of the gastric datasets in Oncomine disclosed that *ZEB1* mRNA was expressed significantly higher in gastric cancer tissues than in normal gastric mucosa (Fig. 10*C*). Kaplan–Meier plotter data analyses uncovered that high *ZEB1* mRNA expression substantially shortened the overall survival time of patients with gastric cancer (Fig. 10*D*). Taken together, these data demonstrated that indisulam degraded ZEB1 in patient gastric tumor tissues and high expression of *ZEB1* deteriorated the progression of gastric cancer.

## Discussion

**Figure 11:**
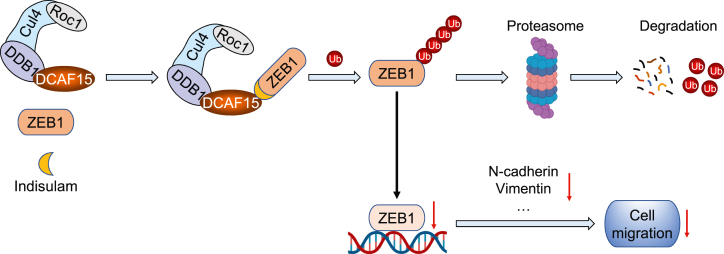
**Proposed model for the inhibitory effect of indisulam on the migration of gastric cancer cells**.

In this work, we first used quantitative proteomics to identify the proteins differentially regulated by the sulfonamide anticancer agent indisulam and discovered that N-cadherin was a significantly downregulated protein in gastric cancer cells. Because N-cadherin is an indicator for EMT and migration of cancer cells (33), we then used the scratch assay to demonstrate that indisulam inhibits the migration of gastric cancer cells. However, the underlying molecular mechanism is unknown. Recent studies reported that indisulam functioned as a molecular glue to recruit neosubstrates for the CRL4 E3 ubiquitin ligases through interacting with the substrate receptor DCAF15, leading to their ubiquitination and degradation (27, 29, 30). Therefore, we thought that indisulam might execute its inhibitory activity on the migration of cancer cells through a similar mechanism. Analysis of protein interaction database revealed that the transcription factor ZEB1, one of the master transcription factors regulating EMT (41), is a potential interactor for DCAF15. Biochemical experiments disclosed that indisulam enhanced the interaction between DCAF15 and ZEB1 and promoted the ubiquitination and proteasomal degradation of ZEB1. Using a cell line–based model, we further demonstrated that indisulam exhibited the inhibitory activity on the migration of gastric cancer cells through reducing ZEB1. This phenomenon is in accordance with the fact that indisulam downregulates ZEB1 in gastric tumor samples and high expression of *ZEB1* mRNA in gastric cancer tissues deleteriously affects patient survival. Taken together, we discovered a new substrate ZEB1 for DCAF15 induced by the molecule glue indisulam and elucidated the molecular mechanism by which indisulam regulates the migration of gastric cancer cells (Fig. 11).

Our previous experiments (32) revealed that alteration of DCAF15 expression did not affect the proliferation of gastric cancer cells in the absence of indisulam. Similarly, our current experiments showed that neither overexpression nor knockdown of *DCAF15* altered the ZEB1 protein level or inhibited the migration of gastric cancer cells. Instead, DCAF15 expression promoted the indisulam-induced downregulation of ZEB1 protein while depletion of DCAF15 eliminated the effect of indisulam on the reduction of ZEB1 protein. However, a previous study in HCC cells (22) revealed that DCAF15 interacted with ZEB1 and triggered its ubiquitination and proteasomal degradation. *DCAF15* knockdown (overexpression) upregulates (suppresses) ZEB1 and then activates (inhibits) EMT. They further discovered that *DCAF15* knockdown promoted HCC cell proliferation and invasion in a ZEB1-dependent manner. Although both our and previous work disclosed the interaction between DCAF15 and ZEB1, the regulation of DCAF15 on ZEB1 is different in two cases. Without indisulam, DCAF15 could significantly downregulate ZEB1 in HCC cells while DCAF15 could hardly regulate ZEB1 in gastric cancer cells. However, indisulam enhances the interaction between DCAF15 and ZEB1 and thus significantly enhanced the ubiquitination and subsequent degradation of ZEB1 in gastric cancer cells. Although we do not know the exact reason, a possible explanation for this discrepancy is that the ubiquitination and degradation of ZEB1 may exhibit cell-type specificity and additional factors, such as adaptor proteins that interact with both DCAF15 and ZEB1, affect the downregulation of ZEB1 by DCAF15.

Interestingly, we discovered that *ZEB1* mRNA level is slightly upregulated by indisulam. However, indisulam still downregulates ZEB1 protein level due to the induced proteasomal degradation. This phenomenon is similar to the effect of indisulam on RBM39 protein and mRNA level reported in our previous work (32). On the one hand, indisulam functions as a molecular glue to promote the ubiquitination and degradation of ZEB1 and RBM39. On the other hand, indisulam elevates their gene expression. This may be a protective feedback effect in cancer cells when the protein level is reduced during drug treatment. It is also possible that indisulam modulates the protein levels of the transcription factors or regulators of these two genes and thus indirectly regulates their gene expression. Nevertheless, the overall effect is the reduction of the protein level, observed both in cell lines and in tumor tissues.

Our experiments disclosed that N-cadherin and vimentin, two EMT markers (42, 43, 44), were also downregulated by indisulam in a time-dependent manner, which is consistent with the fact that ZEB1 is a master transcription factor for EMT (41). Other ZEB1 downstream targets, such as p53, Rb, CDKN1A, and matrix metalloproteases (45), might also contribute to EMT. Previous work and our work utilized different proteomics approaches, including affinity purification and MS (27, 32), pSILAC (46), and label-free quantification, to identify proteins that are differentially regulated by indisulam or indisulam-induced substrates for DCAF15. Experiments with different approaches and different cell lines identified several key proteins, including RBM39 (27, 32), RBM23 (28), PRPF39 (46), and ZEB1. These approaches are complementary with each other, and biochemical methods are effective approaches for the identification of proteins in low abundance. Therefore, a combination of multiple approaches might reveal new molecules that participate in the regulations of the biological function of indisulam in different experimental systems.

However, our work also has its limitations. First, previous studies identified RBM39 as the neosubstrate for DCAF15 upon indisulam treatment through affinity purification or proximity labeling technique and quantitative proteomics (27, 32). Since our previous work revealed that RBM39 mediated the indisulam-inhibited proliferation and this work demonstrated the role of ZEB1 in the regulation of indisulam-inhibited migration of gastric cancer cells, we did not explore the function of RBM39 on indisulam-regulated migration. However, we cannot completely rule out this possibility. Our present study identified several proteins including RBM39 and N-cadherin as the significantly downregulated proteins. Unfortunately, our proteomics studies did not directly detect ZEB1, presumably due to its low abundance in the cell line used for the proteomic work or low sensitivity of the MS instrument used in this work. However, comprehensive studies for the biophysical interactions of ORFeome-derived complexes (BioPlex) discovered the interaction between DCAF15 and ZEB1 (17, 18). Second, we did not identify the lysine residues on ZEB1 that modulate the ubiquitination and stability of ZEB1 in the presence of indisulam. Enrichment of ubiquitinated ZEB1 or ubiquitinated peptides from ZEB1 might facilitate the MS-identification of the ubiquitination sites. In addition, site-directed mutagenesis could be used to discover and validate the modification sites. Third, although we identified N-cadherin as an indisulam-downregulated protein, we did not detect or analyze other downstream targets of ZEB1. Since only a few proteins were identified as differentially regulated proteins, we could not cluster them into specific biological pathways using bioinformatics analysis. However, besides N-cadherin, detailed examination of the differentially regulated proteins also revealed that indisulam significantly reduced the telomere-associated protein RIF1, which plays a key role in the repair of double-strand DNA breaks in response to DNA damage (47, 48), indicating that indisulam might elevate DNA damage and result in the death of cancer cells. It is also possible that there might be additional molecular mechanisms that connect indisulam with N-cadherin. However, we used N-cadherin as a starting point to discover the linkage between indisulam and the key molecule that regulates the indisulam-inhibited migration of gastric cancer cells. Although we elucidated the mechanism by which indisulam regulates the migration of gastric cancer cells by promoting the ubiquitination and degradation of ZEB1, our work could not completely rule out the possibility that indisulam might also regulate other transcription factors and their downstream targets. Nevertheless, based on our work, we can conclude that ZEB1 is, at least, one of the major transcription factors that are responsible for the indisulam-inhibited migration of gastric cancer cells.

In summary, this work demonstrated that indisulam inhibits the migration of gastric cancer cells through degrading the transcription factor ZEB1 and regulating the downstream EMT process, suggesting that indisulam may exhibit antimetastatic activity in gastric cancer. Together with previous discoveries, our work indicates that indisulam, acting as a molecular glue, regulates two biological processes, cell proliferation and migration, of gastric cancer cells through distinct signaling molecules.

## Experimental procedures

### Reagents and antibodies

Indisulam (T4321) was obtained from TargetMol. Anti-FLAG affinity gel (B23102), FLAG peptide (B23112), HA magnetic beads (B26202), and MG132 (S2619) were purchased from Selleck. Puromycin (P8230) was ordered from Solarbio Life Sciences.

Antibodies used in this work were acquired from the following companies: anti-GAPDH antibody (60004-1-Ig), anti-ZEB1 antibody (66279-1-Ig), anti-N-cadherin antibody (220180-1-AP), and anti-Myc antibody (60003-2-Ig) from Proteintech; anti-HA antibody (M180-3) and anti-FLAG (M185-3L) antibody from MBL International; anti-vimentin antibody (ab92547) from Abcam; anti-vinculin antibody (CPA9462) and anti-β-actin antibody (CPA1009) from Cohesion Biosciences; anti-β-tubulin (M1305-2) antibody from HuaAn Biotechnology. Alexa Fluor 488 goat anti-rabbit IgG (A21206) and Alexa Fluor 594 goat anti-mouse IgG (A11005) were ordered from Thermo Fisher Scientific. DAPI (D9542) was from Sigma-Aldrich.

### Construction of plasmids and stable cell lines

DCAF15 and ZEB1 were amplified from cDNA obtained from HEK293 cells. pHBLV-DCAF15-FLAG, pcDNA3.1-FLAG-DCAF15, and pcDNA3.1-HA-ZEB1 were constructed by PCR amplification and cloning using the ClonExpress Ultra One Step Cloning Kit (C115-02, Vazyme). Myc-ubiquitin (Myc-Ub) plasmid was obtained from previous work (32). The pLKO.1-TRC lentiviral vector was used to construct the shRNA plasmids based on a previous method (49). The sense and antisense oligonucleotides (Table S3) were synthesized by GENEWIZ. All constructed plasmids were confirmed by Sanger sequencing (GENEWIZ). Stable cell lines were constructed by using lentiviral infection according to a previously described method (50).

### Cell culture

AGS, HGC27, MGC803, HEK293, and HEK293T cells were maintained in high-glucose Dulbecco's Modified Eagle’s Medium (Gibco) supplemented with FBS (03.U16001DC, EallBio) and penicillin/streptomycin (Gibco). All cell lines were cultured in a humidified incubator containing 5% CO_2_ at 37 °C.

### siRNA transfection

si*NC* (160818) and si*ZEB1* (sense: 5′-GCUGAAAGUCAAGCAAGCAAGCATT-3′, antisense: 5′-UGCUUGCUUGACUUUCAGCTT-3′) were synthesized by Guangzhou RiboBio Co AGS and MGC803 cells were transfected with si*NC* or si*ZEB1* using a lipofectamine RNAiMAX transfection reagent (13778-150, Thermo Fisher Scientific) according to a method described previously (51).

### Affinity purification

Anti-FLAG affinity gel was used to purify FLAG-DCAF15 according to a previously used method (52, 53). Cells expressing FLAG-DCAF15 were washed and lysed with modified RIPA buffer containing freshly prepared protease inhibitor cocktail (B14012, Bimake). Cell lysate was incubated with anti-FLAG affinity gel at 4 °C overnight. The sample was centrifuged briefly and the gel was washed. FLAG-DCAF15 and its interacting proteins were eluted twice with 40 μl modified RIPA buffer containing FLAG peptide (200 μg/ml). Anti-HA magnetic beads were used to purify HA-ZEB1 according to a previous procedure (51). Briefly, anti-HA magnetic beads were incubated with cell lysate at 4 °C overnight with constant shaking and washed. HA-ZEB1 and its interacting proteins were eluted twice with 50 μl 2× SDS sample loading buffer.

### Cycloheximide chase and proteasome inhibition experiments

The CHX chase and proteasome inhibition experiments were carried out as described (54, 55, 56). AGS cells stably expressing sh*NC* or sh*DCAF15* were treated with DMSO or indisulam (10 μM) and CHX (200 μg/ml) for the indicated time. For proteasome inhibition experiments, AGS and MGC803 cells were pretreated with DMSO or indisulam (10 μM) for 48 h and then treated with DMSO or MG132 (10 μM) for 16 h.

### Western blotting analysis

Western blotting analysis was carried out according to a method previously described (51, 57). Cell lysates or affinity-purified samples were mixed with the proper amount of 5× SDS sample loading buffer, boiled, centrifuged, separated by SDS-PAGE, and transferred to PVDF membrane (IPVH00010, Merck Millipore). The membranes were blocked with 5% skimmed milk and incubated with indicated primary and secondary antibodies. Proteins on the membrane were visualized with ECL chemiluminescence reagents (P10300, NCM Biotech), and the chemiluminescent signal was recorded in a Tanon 5200 imaging system.

### Quantitative PCR

Total RNA was isolated with TRIzol (R401-01, Vazyme), and mRNA was reverse-transcribed to cDNA with 5× All-In-One RT MasterMix (G490, ABM). Primers (Table S4) were synthesized by GENEWIZ. qPCR was performed using ChamQ Universal SYBR qPCR Master Mix (Q711-02, Vazyme) in an Applied Biosystems 7500 real-time PCR system.

### Scratch assay

Scratch assay was performed as described (49) to measure cell migration. Briefly, vertical and horizontal lines were marked on the back of the 6-well plates. Gastric cancer cells were cultured with growth medium in these 6-well plates. When cells reached nearly 100% confluence, scratches were made along the marked lines. Cells were washed and cultured in Dulbecco's Modified Eagle’s Medium without FBS. Images were captured under an inverted microscope (IX71, Olympus). The width of the scratches was measured using Image-Pro Plus (Media Cybernetics) at 0 h and 48 h with mock (DMSO) or indisulam (10 μM) treatment. The migration distance within 48 h was calculated by subtracting the width at 0 h for the scratches at the same location.

### Immunofluorescence

The immunofluorescence experiments were performed according to a previous procedure (58, 59). Briefly, HEK293 cells were transfected with FLAG-DCAF15 and/or HA-ZEB1 plasmids for 24 h, washed, fixed, permeabilized, blocked, and incubated with anti-FLAG rabbit monoclonal antibody (1:200) and/or anti-HA mouse monoclonal antibody (1:200) at 4 °C overnight. Cells were further stained with the corresponding secondary antibodies (Alexa Fluor 488 and 594) for 1 h and DAPI for 5 min in the dark at room temperature. The images were taken under a Nikon A1R HD25 confocal microscope.

### Bioinformatics analysis

DCAF15-interacting proteins were analyzed using interactome data from BioGRID (https://thebiogrid.org/). *ZEB1* mRNA expression from gastric adenocarcinoma tissues and gastric mucosa tissues was obtained from Oncomine (https://www.oncomine.org). The relationship between *ZEB1* mRNA expression and patient survival was analyzed using data from Kaplan–Meier plotter (https://kmplot.com/analysis/).

### Sample preparation, MS analysis, and database search

AGS cells expressing DCAF15 from a previous work (32) were treated with DMSO or indisulam (10 μM) for 6 h. The detailed experimental procedures for protein sample preparation, MS analysis, and database search were described in the Supporting Experimental Procedures.

### Collection and treatment of human gastric cancer tissues

Fresh tumor or paratumor tissues from patients with gastric cancer were deidentified and collected from the First Affiliated Hospital of Soochow University. The experimental procedure was approved by the Ethics Committee of Soochow University and performed according to a published procedure (32).

### Statistical analysis

All statistical analyses were performed using GraphPad Prism. Data were displayed as means and standard deviations (mean ± SD). Student’s *t* test (two tailed), two-way ANOVA, and Logrank were used for statistical analyses. ∗*p* < 0.05, ∗∗*p* < 0.01, ∗∗∗*p* < 0.001, ∗∗∗∗*p* < 0.0001, ns: not significant.

## Data availability

The MS data were deposited to the ProteomeXchange Consortium (http://proteomecentral.proteomexchange.org) *via* the iProx (60) partner repository with the dataset identifier PXD036835 (http://proteomecentral.proteomexchange.org/cgi/GetDataset?ID=PXD036835).

## Supporting information

This article contains supporting information.

## Conflict of interest

The authors declare that they have no conflicts of interest with the contents of this article.
