# Supplementary material for: The aryl sulfonamide indisulam inhibits gastric cancer cell migration by promoting the ubiquitination and degradation of the transcription factor ZEB1
Source: J Biol Chem. 2023 Feb 15;299(4):103025. doi: 10.1016/j.jbc.2023.103025 (PMC10040736; doi:10.1016/j.jbc.2023.103025)
Supplement: Supporting information [file mmc1.pdf]

Supporting information

Supporting Experimental Procedures

Sample preparation and MS analysis

AGS cells expressing DCAF15 were treated with DMSO or indisulam (10 μM) for 6 h, washed three times with ice-cold PBS, and collected. Cells were lysed with brief sonication in the lysis buffer containing 8 M urea, 10 mM HEPES (pH 8.0), 150 mM NaCl, and freshly added protease inhibitor cocktail. Proteins from cell lysates were reduced, alkylated, and precipitated with cold acetone. The precipitate was briefly dried in air and resuspended in 8 M urea with 10 mM HEPES (pH 8.0) and 150 mM NaCl. The resuspended proteins were digested with Lys-C (1:50 enzyme:protein ratio) for 3 h and trypsin (1:25 enzyme:protein ratio) for 16 h at 37 °C after diluting urea to 2 M with 10 mM HEPES (pH 8.0). The digests were acidified with trifluoroacetic acid and centrifuged to remove the pellet. Peptides were desalted with C18 ZipTip, vacuum-dried, and resuspended in 0.1% formic acid.

Peptide samples were analyzed by an Orbitrap Fusion Lumos Tribrid mass spectrometer (MS) with an electrospray ionization inlet (Thermo Fisher Scientific). Samples were separated on a C18 analytical column through a nanoscale HPLC with solvent A of 0.1% formic acid and solvent B of 80% acetonitrile and 0.1% formic acid. The HPLC solvent B gradient was as follows: 5% in 5 min, 5%–40% for 70 min, 40%–44% for 5 min, 44%–100% in 5 min, and 100% for 5 min. MS and MS/MS spectra were acquired in a positive-ion mode with an automated Xcalibur data acquisition system. Each precursor ion was analyzed twice in 60 s. The resolution for the precursor ion was set to 120,000 at 200 m/z and the isolation window of the selected precursor ion for MS/MS analysis was set to 1.6 m/z. Each sample was analyzed for four technical replicates and three biological replicates were performed.

Database search

All raw MS data files were searched with Proteome Discoverer (version 2.2, Thermo Fisher Scientific) against the UniProt human protein database (www.uniprot.org) concatenated with common contaminants and the decoy database. The cysteine carbamidomethylation was set as fixed modification and methionine oxidation and N-terminal acetylation were set as variable

modifications. Enzyme specificity was set to trypsin and a maximum of two missed cleavages per peptide were allowed. The mass tolerance for precursor ions and fragment ions was set to 10 ppm and 0.02 Da, respectively. The 1% false discovery rate (FDR) at both peptide and protein levels was applied for the analysis. Relative protein quantification was performed using the label-free quantification (LFQ) incorporated in the Proteome Discoverer. The common contaminants were removed and proteins with at least three quantification data were retained. The zero value was replaced by a random number, which was calculated from a normal distribution with a width of 0.3 and a downshift of 1.8 defined by Perseus software (version 1.6.5.0). The *P*-value was calculated by performing two-sample Student's *t*-test. Perseus was used to calculate $-\text{Log}_{10}$ (*P*-value) and Log_2 (Indisulam/DMSO) for the construction of the volcano plot.

Supporting Tables

Table S3. Oligonucleotides used for the construction of sh*RNA* plasmids.

Oligonucleotides for sh*RNA*	Sequence
sh*DCAF15*#1 sense	CCGGGCGTGTCCCTCAAGAACATTGCTCGAGCAATGTTCTTGAGGGACACGCTTTTTG
sh*DCAF15*#1 antisense	AATTCAAAAAGCGTGTCCCTCAAGAACATTGCTCGAGCAATGTTCTTGAGGGACACGC
sh*DCAF15*#2 sense	CCGGGGACACTGACTCCAACTACCTCTCGAGAGGTAGTTGGAGTCAGTGTCCTTTTTG
sh*DCAF15*#2 antisense	AATTCAAAAAGGACACTGACTCCAACTACCTCTCGAGAGGTAGTTGGAGTCAGTGTCC

Note: underlined oligonucleotides are the targeting sequences.

Table S4. Primers used for qPCR.

qPCR Primers	Sequence
DCAF15-F	GTGCGTGTCCCTCAAGAACAT
DCAF15-R	CGTCCTGGAATAGCCGAACC
ZEB1-F	ACTCTGATTCTACACCGC
ZEB1-R	TGTCACATTGATAGGGCTT
N-cadherin-F	CATCCCTCCAATCAACTTGCC
N-cadherin-R	ATGTGCCCTCAAATGAAACCG
GAPDH-F1	TGCACCACCAACTGCTTAGC
GAPDH-R1	ACAGTCTTCTGGGTGGCAGTG
GAPDH-F2	GAGTCAACGGATTTGGTCGT
GAPDH-R2	TTGATTTTGGAGGGATCTCG

Supporting Figures

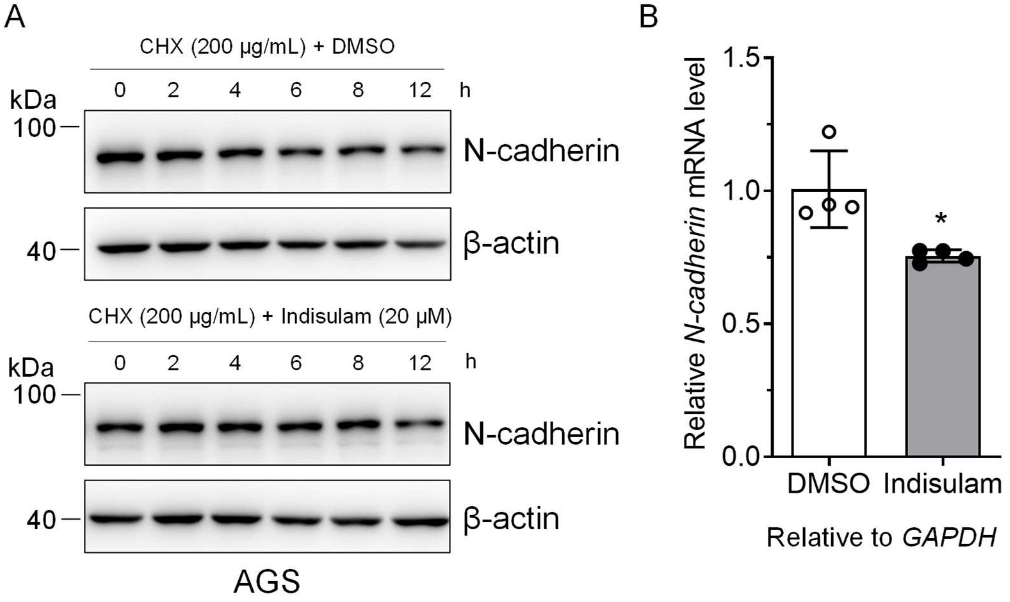

Figure S1. Indisulam does not significantly affect the degradation of N-cadherin protein but reduces *N-cadherin* mRNA levels. (A) AGS cells were pretreated with DMSO or indisulam (20 μM) for 72 h, then treated with DMSO or indisulam (20 μM) and CHX (200 μg/mL) for the indicated time. Cell lysates were immunoblotted for the indicated proteins. (B) AGS cells were treated with indisulam (20 μM) for 72 h and qPCR was performed to analyze the relative mRNA levels. *GAPDH* was used as the loading control. The points on the bar graph represented the data from four technical replicates. The experiments were performed twice and similar results were obtained.

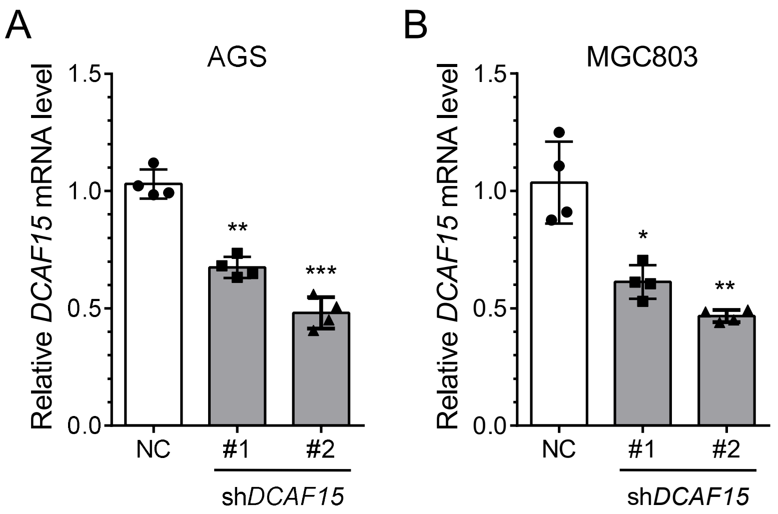

Figure S2. *DCAF15* mRNA levels in *DCAF15*-knockdown stable AGS and MGC803 cells. (A-B) AGS and MGC803 cells were infected with the control or sh*DCAF15* lentiviral particles and selected by puromycin for two weeks to obtain stable cell lines. Relative *DCAF15* mRNA levels were determined by qPCR. *GAPDH* was used as the loading control. Mean ± SDs (n = 4), Student's *t*-test, *: $P < 0.05$, **: $P < 0.01$, ***: $P < 0.001$. Similar results were obtained from two biological replicates.

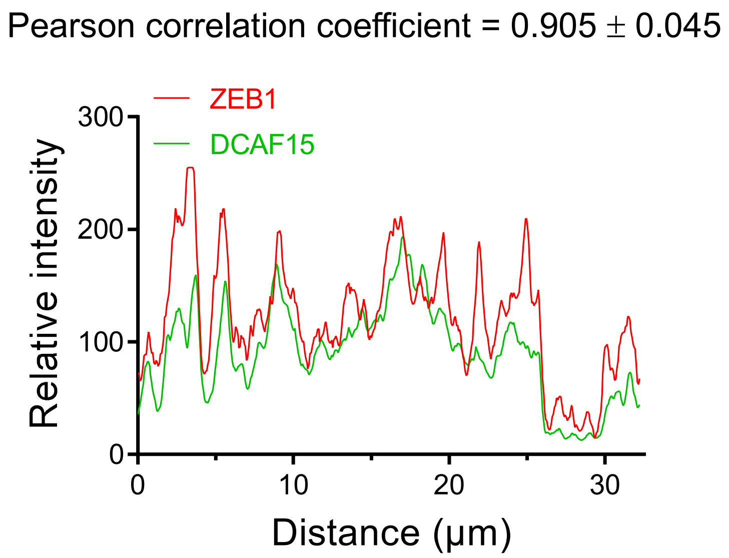

Figure S3. Correlation analysis of ZEB1 and DCAF15 colocalization. The Pearson correlation coefficient was 0.905 ± 0.045 (Mean ± SD). The data were obtained from three cells.

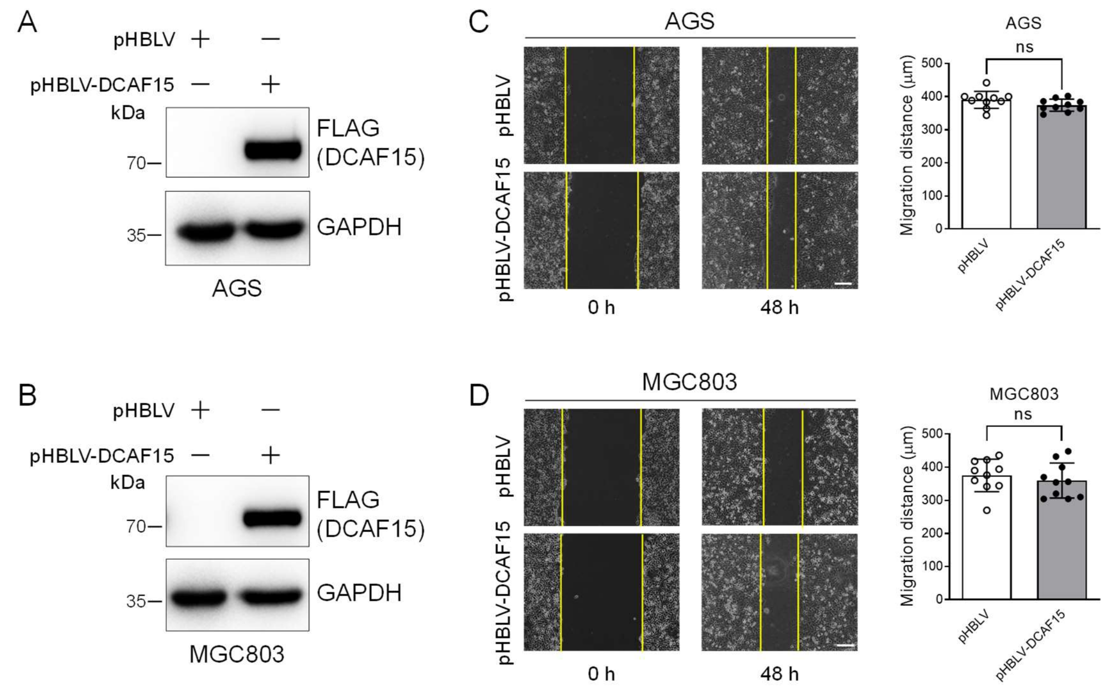

Figure S4. Expression of DCAF15 does not affect the migration of AGS and MGC803 cells in the absence of indisulam. (A-B) AGS and MGC803 cells were infected with the pHBLV or pHBLV-DCAF15 lentiviral particles and selected with puromycin for two weeks to obtain stable cell lines. DCAF15 expression was evaluated by immunoblotting analysis. (C-D) Scratch assay was performed to measure cell migration. Scale bar: 200 μm. Student's *t*-test, Mean ± SDs (n = 10), ns: not significant. These data were obtained from three biological replicates.

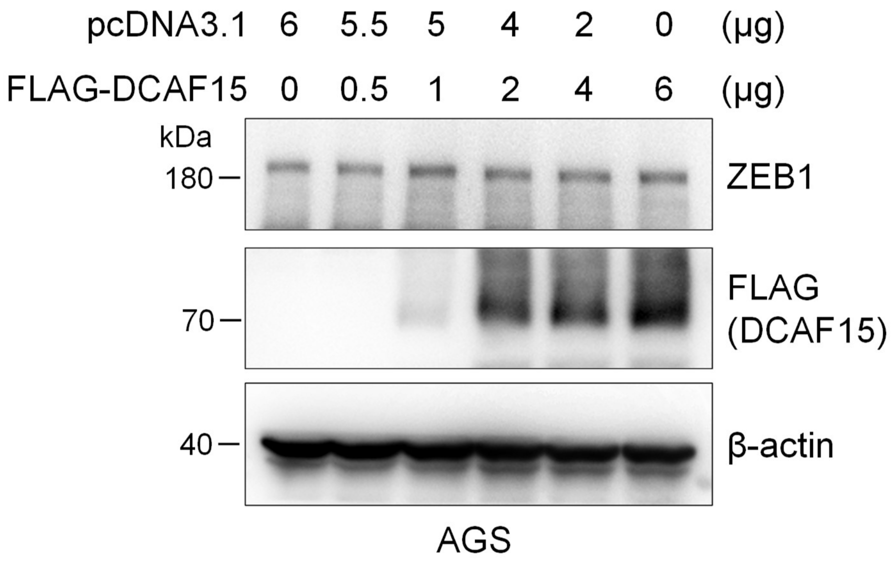

Figure S5. Gradient transfection of DCAF15 does not affect the ZEB1 protein level. AGS cells were transfected with different amount of pcDNA3.1 or

FLAG-DCAF15 plasmid in 6-well plates for 60 h. Cell lysates were immunoblotted with the indicated antibodies. pcDNA3.1 was used as the control and balancing plasmid.

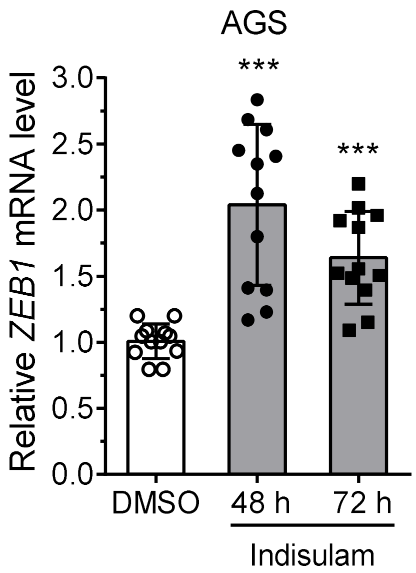

Figure S6. Indisulam elevates the *ZEB1* mRNA level in AGS cells. AGS cells were treated with indisulam (10 μM) for 48 or 72 h and the *ZEB1* mRNA was determined by qPCR. *GAPDH* was used as a loading control. Mean ± SDs (n = 12), Student's *t*-test, ***: $P < 0.001$. The data were obtained from three biological replicates, each with four technical replicates.

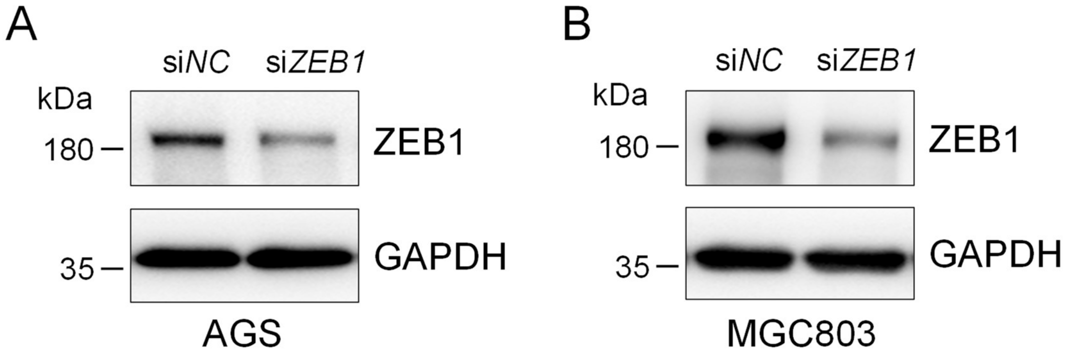

Figure S7. Evaluation for *ZEB1* knockdown efficiency in AGS and MGC803 cells. AGS (A) and MGC803 (B) cells were transfected with si*NC* (negative control) or si*ZEB1* using lipofectamine RNAiMAX for 48 h. Cell lysates were immunoblotted with the indicated antibodies.

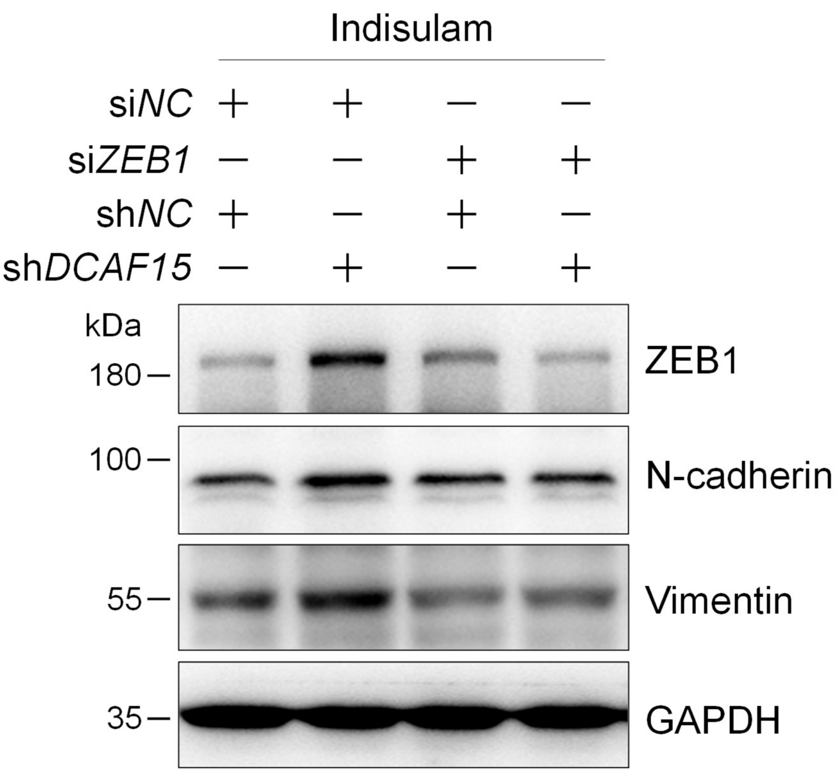

Figure S8. ***ZEB1*** **knockdown eliminates the DCAF15-mediated effect of indisulam on the expression of epithelial to mesenchymal transition (EMT) markers.** The sh*NC* and sh*DCAF15*-expressing stable AGS cells were transfected with the control (*NC*) and *ZEB1* specific siRNA with lipofectamine RNAiMAX. At 24 h after transfection, cells were treated with indisulam (10 μM) for 48 h. The cell lysates were subjected to immunoblotting analysis.

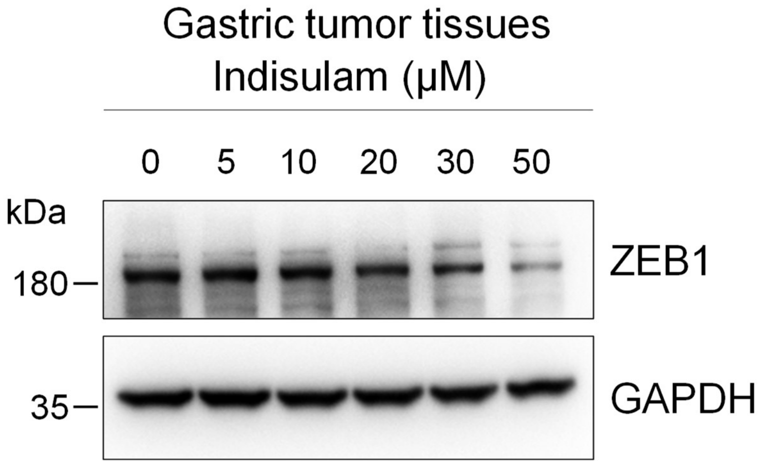

Figure S9. Indisulam downregulates the protein level of ZEB1 in gastric cancer tissues. Gastric cancer tissues were treated with the indicated concentration of indisulam for 72 h, and ZEB1 protein was analyzed by immunoblotting.

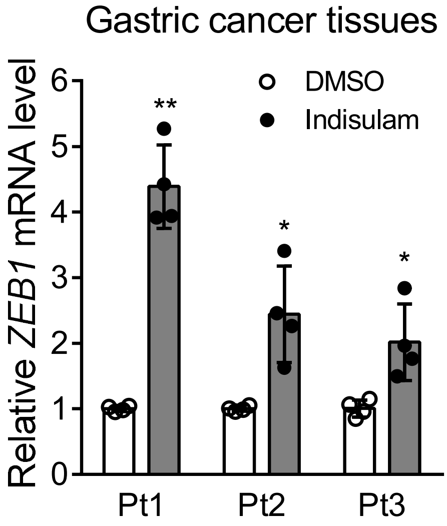

Figure S10. Indisulam elevates the *ZEB1* mRNA level in gastric cancer tissues. Gastric cancer tissues were treated with indisulam (50 μM) for 72 h, and the *ZEB1* mRNA level was determined by qPCR.
